# Ultimate Load-Carrying Ability of Rib-Stiffened 2024-T3 and 7075-T6 Aluminium Alloy Panels under Axial Compression

**DOI:** 10.3390/ma14051176

**Published:** 2021-03-02

**Authors:** Ján Slota, Andrzej Kubit, Tomasz Trzepieciński, Bogdan Krasowski, Ján Varga

**Affiliations:** 1 Department of Technologies and Materials, Institute of Technology and Materials Engineering, Technical University of Košice, Mäsiarska 74, 040 01 Košice, Slovakia; jan.slota@tuke.sk (J.S.); jan.varga@tuke.sk (J.V.); 2 Department of Manufacturing and Production Engineering, Rzeszow University of Technology, al. Powst. Warszawy 8, 35-959 Rzeszów, Poland; 3 Department of Materials Forming and Processing, Rzeszow University of Technology, al. Powst. Warszawy 8, 35-959 Rzeszów, Poland; 4 Department of Mechanics and Machine Building, Carpatian State School in Krosno, ul. Żwirki i Wigury 9A, 38-400 Krosno, Poland; b_krasowski@wp.pl

**Keywords:** aluminium alloy, axial compression test, incremental sheet forming, load-carrying capacity, rib-stiffened panel

## Abstract

Stringer-stiffened panels made of aluminium alloys are often used as structural elements in the aircraft industry. The load-carrying capacity of this type of structure cannot relieve the reduction in strength in the event of local buckling. In this paper, a method of fabrication of rib-stiffened panels made of EN AW-2024-T3 Alclad and EN AW-7075-T6 Alclad has been proposed using single point incremental forming. Panels made of sheets of different thickness and with different values of forming parameters were tested under the axial compression test. A digital image correlation (DIC)-based system was used to find the distribution of strain in the panels. The results of the axial compression tests revealed that the panels had two distinct buckling modes: (i) The panels buckled halfway up the panel height towards the rib, without any appreciable loss of rib stability, and (ii) the rib first lost stability at half its height with associated breakage, and then the panel was deflected in the opposite direction to the position of the rib. Different buckling modes can be associated with the character of transverse and longitudinal springback of panels resulting from local interaction of the rotating tool on the surface of the formed ribs.

## 1. Introduction

Aluminium alloy sheets are materials commonly used in the aviation and aerospace industries due to their favourable strength properties combined with low density. Of the aluminium alloys available, age-hardenable 7000 series alloys with a high zinc content are increasingly used. Despite exhibiting high strength (although mainly in the hardened state), the plastic working of these alloys is limited [1]. Along with increasing the forming temperature, the plasticity of the aluminium alloys and the quality of the parts obtained from them are visibly improved. Another problem with these alloys is high springback [2]. Due to the low Young’s modulus of aluminium alloys compared to steel (70 GPa compared with 210 GPa), the springback of parts made of high-strength aluminium is greater than that of steels with comparable strength properties.

The 2000 series aluminium alloys contain several percent of copper and additives of magnesium and manganese. They have high strength and medium resistance to corrosion. The 2000 series aluminium alloy grades are good for parts requiring good resistance to temperatures up to 150 °C. With the exception of grade 2219, these aluminium alloys have limited weldability [3,4], but some of the alloys in this series have excellent machinability [5,6]. EN AW-2024 [7] and EN AW-7075 [7] aluminium alloys are the most popular grades used in the aerospace industry for skins and wing spars, compressor blades, fan discs, gears and shafts, fuse parts, regulating elements of valves, and other parts of commercial aircraft and aerospace vehicles.

Due to their good stiffness properties, thin-walled aluminium alloy structures are widely used in the automotive and aircraft industries. Thin-walled plates used in the construction of aircraft are mainly subjected to compressive load. Under compression load, the structure may lose stability, thus reducing its load capacity. When the post-critical equilibrium path is stable and loss of stability occurs in the elastic range, such elements can operate in a post-critical range [8,9]. In the elastic range, the buckled portions of the plate shed load and become ineffective in resisting further loading, while in the portions of the plate close to the supports, the out of plane buckling is diminished, and these parts have post-buckling reserves of strength and stiffness [10]. For a plate without imperfections the post-buckling axial stiffness drops immediately upon buckling, and thereafter reduces still further as loading increases.

Aircraft stringer-stiffened components and fuselage components are joined in a metallic structure by means of welding or riveting to form a complete wing and fuselage structure [11]. The rib-stiffened panels can be formed integrally, for example by using Single Point Incremental Forming (SPIF) [12,13]. Rib-stiffened panels have many advantages that include cost savings through reductions in tooling, assembly labour, and manufacturing time [14,15].

As buckling is the dominant limitation for thin-walled stringer-stiffened structures subjected to in-plane loads, it is important to have a good understanding of the phenomenology of structural response. Research on the load-carrying capacity of stringer-stiffened panels is carried out quite widely. Wilckens et al. [16] analysed buckling and post buckling behaviour of stringer-stiffened panels made of carbon fibre reinforced polymer under axial compression loading. The results of numerical modelling based on the finite element method matched well with the experimental results in terms of buckling stiffness and critical load. Kubit et al. [17] tested a fibre metal laminate-based stringer-stiffened thin-walled structure in a uniaxial tensile test, tensile/shear test, and peel drum test. The stringers were tested at temperatures corresponding to real aircraft operations, i.e., +80 °C, room temperature, and −60 °C. It was found that the temperatures do not affect the mechanical properties of the structures tested. Su et al. [18] analysed the structural behaviour of Ti6Al4V titanium alloy-stiffened panels under shear load experimentally and by numerical analysis. They found that after the initial local buckling on the skin, the buckling mode jumps several times with the increase of load. The results of experiments on the impact damage of reinforced composite plates conducted by Li et al. [19] showed that the energy under the low-velocity impact test causes different degrees of damage including debonding of the skin and stiffeners, and internal delamination of the skin. The stiffness and shape of the cross-section and of a stringer play an important role in its stability under axial load [20,21]. Mo et al. [22] analysed buckling and post-buckling behaviour of stringer-stiffened composite flat panels subjected to axial compression. It was found that a hat-stringer flat panel exhibited large load capacity after initial buckling. Shao and He [23] investigated the carrying capacity of a stiffened thin-walled composite plate under shear load, and the results indicated that skin thickness has a key effect on the carrying-capacity of the panel. The effect of stringer height and stringer thickness on buckling load was studied by Su et al. [18]. They concluded that an increase of stringer height has no influence on the initial buckling mode.

Stiffened panels are widely adopted to save structural weight and greatly reduce the use of materials. Stringers on the stiffened panels carry out and transfer loads. There are different shapes of stringers that are commonly riveted to the skin, i.e., C-shape [24], hat-shape [25,26], I-shape [24,27], J-shape [28], L-shape [24,29,30], T-shape [29,31], and Z-shape [32,33]. Due to the advantages of high stability, superior damage tolerance [34], and light weight [35,36], grid stiffened panels have been applied in the aircraft and aerospace industries. Three main type of grids are used in practical applications [37,38,39], i.e., angle-grid, isogrid, and orthogrid. In this manuscript a novel type of rib-stiffened structure is proposed that is fabricated using SPIF. Aluminium alloy sheets (EN AW-2024-T3 and EN AW-7075-T6), widely used in aircraft industry, were selected as a test material. The load-carrying capacity of panels was tested in an axial compression test. Moreover the fracture morphology of the buckled panels and distributions of strains in the ribs were examined.

## 2. Materials and Methods

### 2.1. Material

Rib-stiffened panels were fabricated from aluminium alloy sheets:

- 0.4-mm thick EN AW-2024-T3 Alclad,
- 1-mm-thick EN AW-2024-T3,
- 0.8-mm thick EN AW-7075-T6 Alclad.

These alloys are commonly used in the aircraft industry. To assure high corrosion resistance, these alloys are supplied in clad form with a thin surface layer of high purity aluminium (Alclad) (Kaiser Aluminum, Spokane, WA, USA). EN AW-2024 is an aluminium alloy whose main alloying component is copper (Table 1). It is used in applications requiring a high strength-to-weight ratio as well as good fatigue resistance. The EN AW-7075-T6 aluminium alloy with zinc and magnesium as the main alloying elements (Table 1) is gaining more and more popularity in aircraft applications due to its unique physical and mechanical properties: Low density, high strength, good plasticity, very high fatigue resistance, and satisfactory corrosion resistance. The upper skins are made of EN AW-7075-T6, because the critical requirement is high compressive strength. Shear webs and ribs are often made of both EW AW-2024-T4 and EN AW-7075-T6 aluminium alloys. Although EN AW-7075-T6 is stronger than EN AW-2024-T3 alloy, structures fabricated in EN AW-7075-T6 have less weight than is possible in an EN AW-2024-T3 structure with equivalent performance. The mechanical properties of the materials of the test sheets are shown in Table 2.

### 2.2. Forming Method

**Figure 1:**
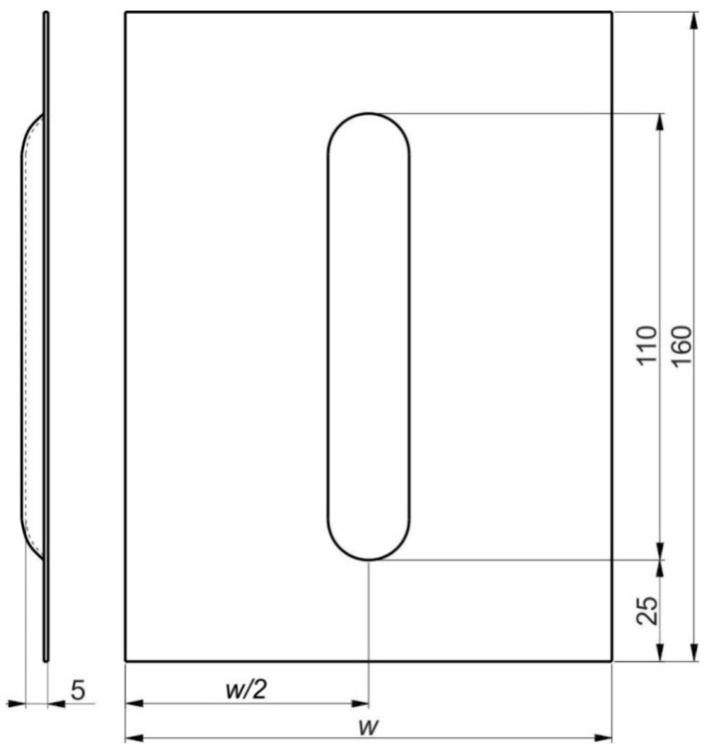
Dimensions (in mm) of a rib-stiffened panel fabricated using Single Point Incremental Forming (SPIF).

**Figure 2:**
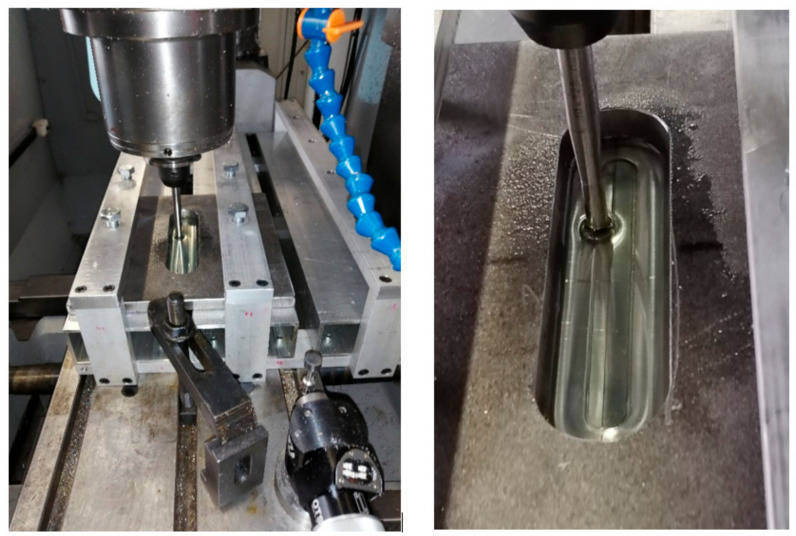
Device for incremental forming mounted in the table of a numerically controlled TM-1P vertical milling machine.

160-mm-long sheets were used to form the ribbed panels (Figure 1). Samples from 0.4-mm-thick EN AW-2024-T3 Alclad and 0.8-mm-thick EN AW-7075-T6 Alclad sheets had a width of w = 120 mm. 1-mm-thick EN AW-2024-T3 sheets were w = 100 mm wide. A HS2-9-2 (1.3348) steel pin with a rounded tip with a radius of r = 3.5 mm was used for forming. Mannol FWD Getriebeoel SAE 75W-85 gear oil (Mannol, Wedel, Germany) was used to reduce the coefficient of friction between the tool and the sheet. The basic properties of the oil that was used were provided by the manufacturer and are as follows: Density 879 kg/m^3^ (at 15 °C), flash point 210 °C, pour point −45 °C, viscosity at 40 °C 72.4 mm^2^/s, viscosity index 157. The device (Figure 2) for incremental forming was mounted in the table of a numerically controlled TM-1P (Hass Automation, Oxnard, CA, USA) vertical milling machine.

**Figure 3:**
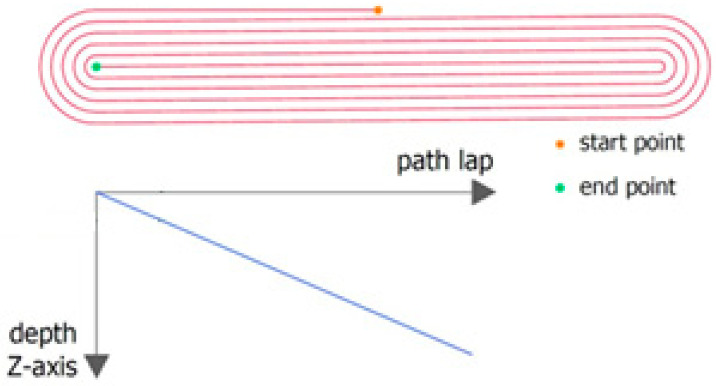
Tool strategy.

The sheet was placed in the tool and clamped at the edges between the two plates using screws. During forming, the tool moves in accordance with the path designed in the control program. As a result of this process, a rib-stiffened panel is obtained, which is formed due to the accumulated, localized plastic deformation resulting from the interaction of the spherically ended tool on the sheet metal. SPIF gives the best results when using a continuous spiral-shaped toolpath (Figure 3) [16]. To prepare the control program, the Edge CAM program (Hexagon AB, Stockholm, Sweden) was used.

Tests on the forming of U-shaped ribs were carried out with a constant rotational speed of the tool equal to v = 96 rpm and a feed rate of f = 800 mm/min. 

The values of tool rotational speed *v* and feed rate f were determined based on a series of trial and error experiments. The rotational speed of the tool and feed rate were considered at three levels:

- v = 18, 96, and 174 rpm,
- f = 400, 800, 1200 mm/min.

A feed rate in the range of 400–800 mm/min did not have a significant effect on the ability to form the ribs. However, at the slower feed rate, the forming process is less economic. When forming with a feed rate of 1200 mm/min, an intensification of machine vibrations was observed due to the rapid change in the direction of movement of the forming tool in the corners of the ribs. As with the feed rate, there is no negative effect of tool speed v = 18 rpm and v = 96 rpm on the ability to fabricate the ribs. Increasing the tool rotational speed to v = 174 rpm in combination with feed rates f = 800 mm/min and f = 1200 mm/min caused a significant deterioration of the surface roughness of the ribs. So, the synergy of the values of v = 96 rpm and f = 800 mm/min is economically and technologically justified.

**Figure 4:**
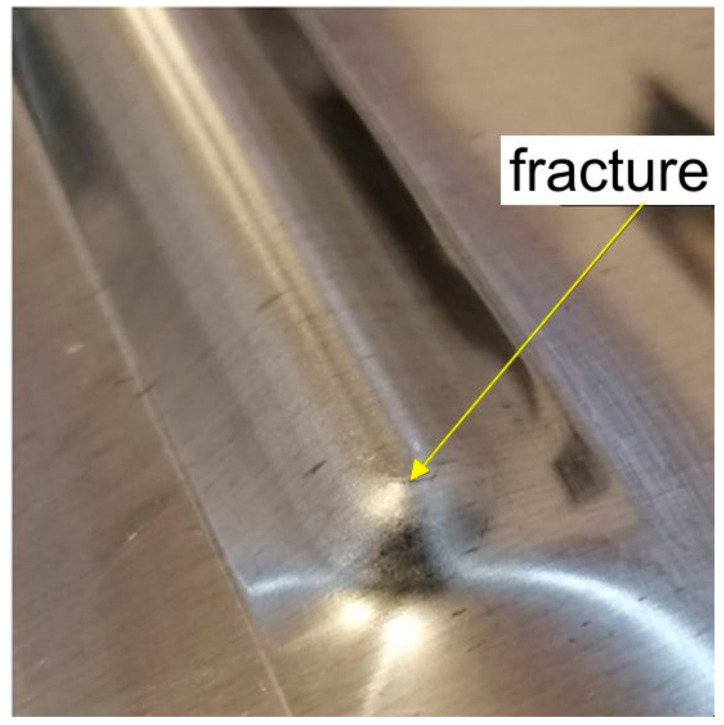
Fracture in the corner of the rib formed in 0.4-mm-thick EN AW-2024-T3 sheet.

In the tests, the ribs were formed with a vertical pitch of the tool a_p_ = 0.2 mm and a_p_ = 0.5 mm. However, the forming of 0.4-mm thick EN AW-2024-T3 Alclad sheets caused the deformation limit of the sheet material to be exceeded and the formation of a crack in the material (Figure 4). The corner of the rib where a complex stress state exists was the area requiring the most effort during forming. To ensure comparable strength tests of all panels, the values of vertical pitches of a_p_ = 0.2 and a_p_ = 0.4 mm were established. In these conditions all the panels were successfully formed.

### 2.3. Axial Compression Test

**Figure 5:**
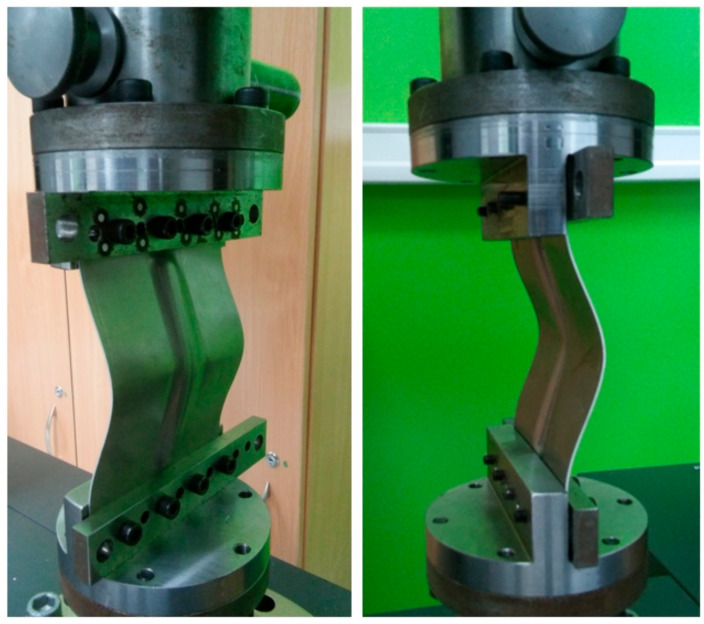
Rib-stiffened panel mounted in the grips of the tensile testing machine.

The rib-stiffened panels were subjected to an axial compression test on a Z100 testing machine (Zwick/Roell, Ulm, Germany) with a maximum capacity of 10 kN. Holes were drilled at both ends of the test panels to enable it to be fixed in the clamps attached to the grippers of the testing machine (Figure 5). The compression test, conducted at 24 °C, consisted in compressing the panel with a gently increasing force of 25 N per second. As a result of the load, the test panel was destroyed as the compressive force increased due to its loss of stability. 

### 2.4. Analysis of Rib Deformation

The strain distribution of the sheet material in the area of the ribs was determined using the Argus (GOM Gmbh, Braunschweig, Germany) non-contact material-independent measuring system based on digital image correlation. The Argus uses a 12-megapixel camera sensor and is mainly used to measure deformations in the processes of sheet metal forming in order to assess the quality of sheet metal products, or to conduct a comparative analysis of mathematical and physical modelling [41,42,43]. When measuring drawpieces, the position of the camera recording the image is changed in at least two planes [44]. In each camera position, the object relative to the camera is rotated by a specific angle. The measurements of sheet strains using the Argus system were performed in the central part of the panel, near the shaped rib.

**Figure 6:**
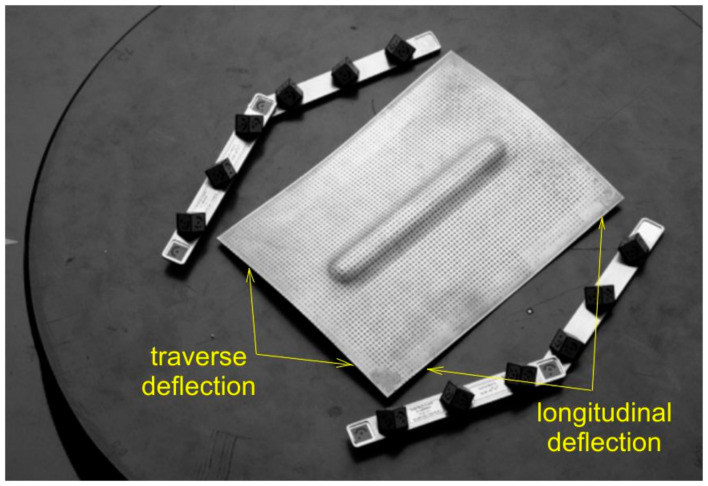
The view of the panel with grid of points.

The measurement was carried out by referring to the change in the position of black points, with a diameter of 1 mm, which were etched onto the surface of the panel before the SPIF process (Figure 6). The distance between the centres of the etched points was 2 mm. Based on the change in position of the points after the forming process, Argus was able to capture the 3D coordinates of the surface presented in a fine resolution mesh. Thanks to the use of a shadeless tent, almost all reflections from the panel surface were eliminated.

### 2.5. Fracture Morphology

The morphologies of the fracture surfaces of the panels were examined using an S-3400 scanning electron microscope (SEM) Phenom ProX (Nanoscience Instruments, Phoenix, AZ, USA). 

## 3. Results

### 3.1. Static Compression Tests

**Figure 7:**
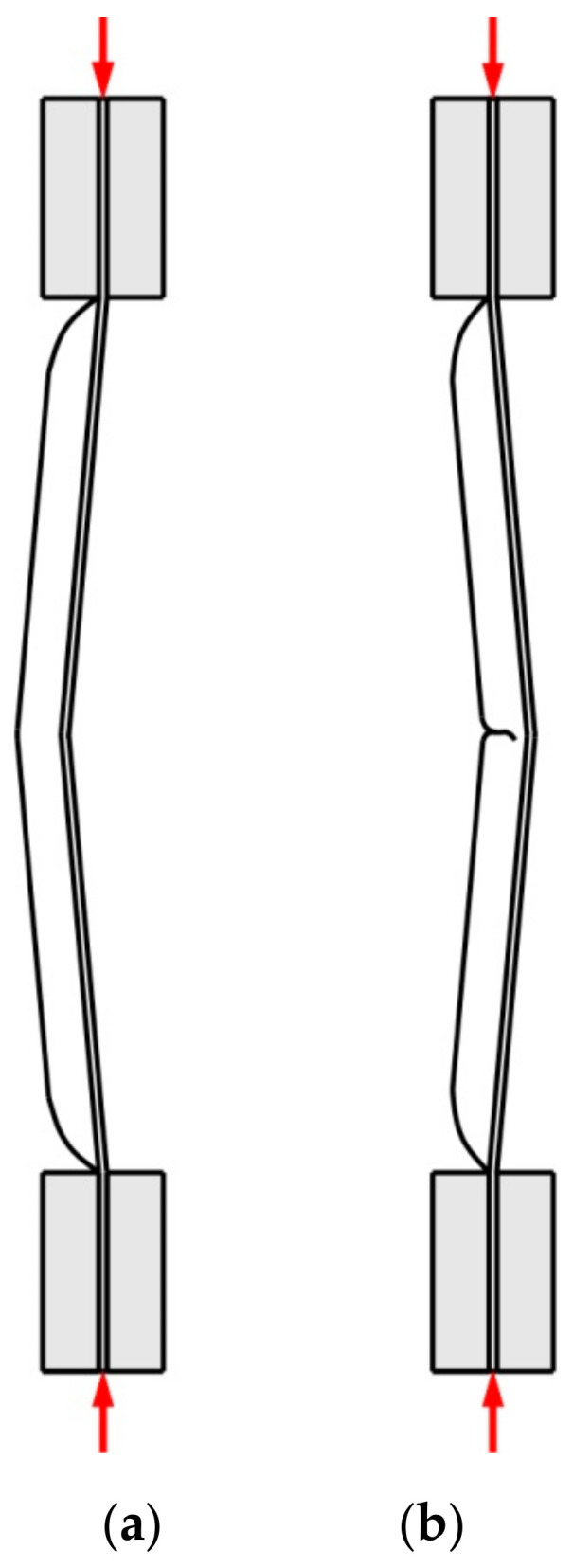
Modes of panel buckling: (**a**) Mode A—the panels buckled halfway up the panel width towards the rib, without any appreciable loss of rib stability and (**b**) Mode B—the rib first lost stability at half height, which was associated with breakage of the rib, and then the panel was deflected in the opposite direction to the position of the rib

As a result of the load, the test panels were damaged as the compressive force increased due to their loss of stability. After plastic deformation of the rib, symmetrical deformation of the flat part of the panel (Figure 6) was observed. Plastic deformation and destruction of the panel under axial compression took place according to two modes: Mode A—the panels buckled halfway up the panel width towards the rib, without any appreciable loss of rib stability (Figure 7a), and mode B—the rib first lost stability at half height, which was associated with breakage of the rib, and then the panel was deflected in the opposite direction to the position of the rib (Figure 7b).

**Figure 8:**
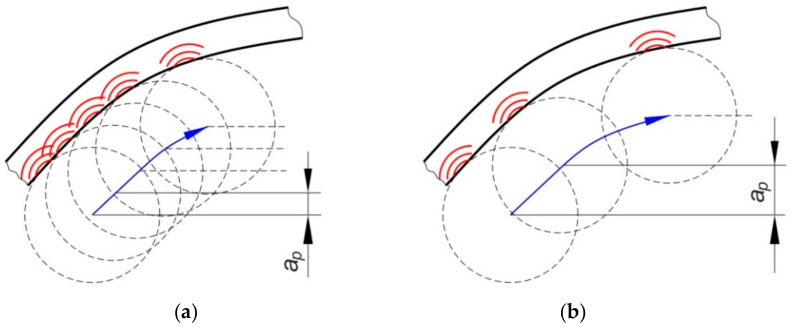
Effect of vertical pitch on the manner of interaction of the tool with the sheet metal at (**a**) small and (**b**) high value of vertical pitch.

Such modes of buckling behaviour can be associated with the amount of deflection of the panels in relation to their springback after the forming process due to interaction of the tool tip with the surface of the rib. During the small vertical pitch of the tool, the intense action of the tool on the rib surface (Figure 8a) caused the sheet to be pulled from the area under the blank holder. Of course, this mechanism only takes place in the vicinity of the middle part of the longitudinal edge of the rib. The springback mechanism is associated with high residual stress in the sheet, which may affect the behaviour of the sheet during the axial compression test.

The other mechanism which could have had a lesser impact on the amount of springback was the work hardening of the ribs. In the conditions under which the panels were formed at a vertical pitch a_p_ = 0.2 mm, the springback of the flat surface of the panels (Figure 6) was greater. In these conditions, the failure of the panel proceeded under buckling mode A. The ribs shaped in this manner showed a lower longitudinal deflection after springback (Figure 6) than profiles shaped at a_p_ = 0.4 mm (Figure 8b). The high value of the vertical pitch meant that there was less material in the rib. The small value of the vertical pitch of the tool resulted in a less intense interaction of the tool on the sheet. Consequently, the observed deflection of the flat surface of the panels conserved less material and therefore the panel was more susceptible to free bending, which led to the formation of buckling mode B.

**Figure 9:**
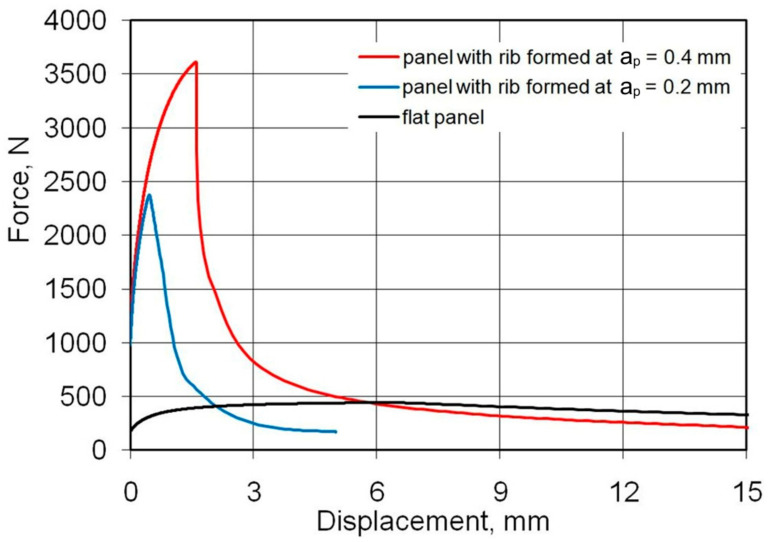
Load-displacement curves of the panels fabricated from 0.4-mm-thick EN AW-2024-T3 Alclad panels subjected to compression load.

**Figure 10:**
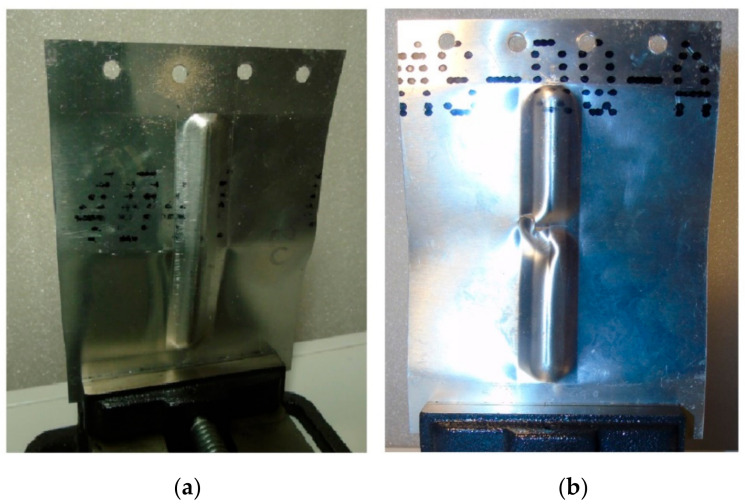
Buckling mode of 0.4-mm-thick EN AW-2024-T3 Alclad panel: (**a**) Mode A—the panels buckled halfway up the panel width towards the rib, without any appreciable loss of rib stability; (**b**) Model B—the rib first lost stability at half height, which was associated with breakage of the rib, and then the panel was deflected in the opposite direction to the position of the rib.

Figure 9 shows the load-displacement curves of the panels fabricated from 0.4-mm-thick EN AW-2024-T3 Alclad sheets subjected to a compression load. The highest compressive strength is shown by a sample that has buckled in the direction opposite to the shaped rib (mode B, Figure 10b). These conditions correspond to the formation of the rib at a_p_ = 0.4 mm. Such a method of deformation caused a 52% increase in maximum force for the panel buckling in comparison with the load on the panel formed at a_p_ = 0.2 mm. Forming the stiffening ribs at a decreased vertical pitch of from a_p_ = 0.4 mm to a_p_ = 0.2 mm increased the destructive force by 763% and 502%, respectively, in relation to the compression of a flat, non-ribbed panel. In the case of both rib-stiffened panels, the loss of stability occurred rapidly, just after reaching the maximum value force. The loss of stability of the panel formed at a_p_ = 0.2 (Figure 10a) occurred at the displacement of the grippers equals of 0.46 mm. Forming of the panel with a_p_ = 0.4 provided greater stiffness of the panel—loss of stability occurred at 1.6 mm.

**Figure 11:**
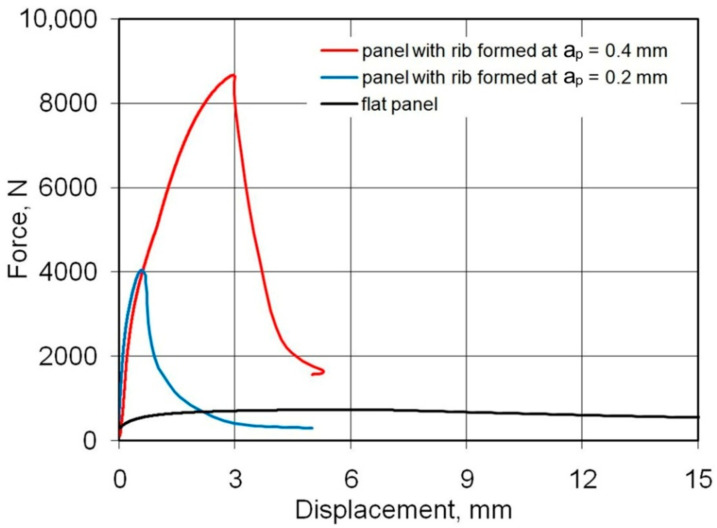
Load-displacement curves of the panels fabricated from 0.8-mm-thick EN AW-7075-T6 Alclad panels subjected to compression load.

**Figure 12:**
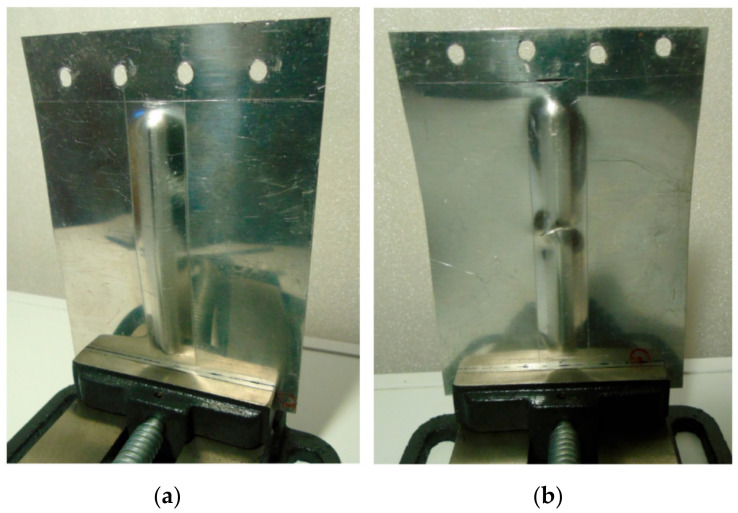
Buckling mode of 0.8-mm-thick EN AW-7075-T6 Alclad panel: (**a**) Mode A—the panels buckled halfway up the panel width towards the rib, without any appreciable loss of rib stability; (**b**) Mode B—the rib first lost stability at half height, which was associated with breakage of the rib, and then the panel was deflected in the opposite direction to the position of the rib.

Figure 11 shows the load-displacement curves of the 0.8-mm-thick EN AW-7075-T6 Alclad panels. The non-ribbed specimen buckled freely with a maximum force of 738 N. As in the case of panels made of 0.4-mm-thick EN AW-2024-T3 Alclad panels, a maximum force of 8674 N was recorded for the panel formed at a_p_ = 0.4 mm, which was destroyed at buckling mode B (Figure 12b). The panel formed in a_p_ = 0.2 mm was rapidly buckled halfway up the panel height towards the rib (Figure 12a) with a maximum force of 3874 N. An increase in the value of the load-carrying capacity of the panels resulted from the change of forming conditions from a_p_ = 0.2 mm to a_p_ = 0.4 mm was 225%.

**Figure 13:**
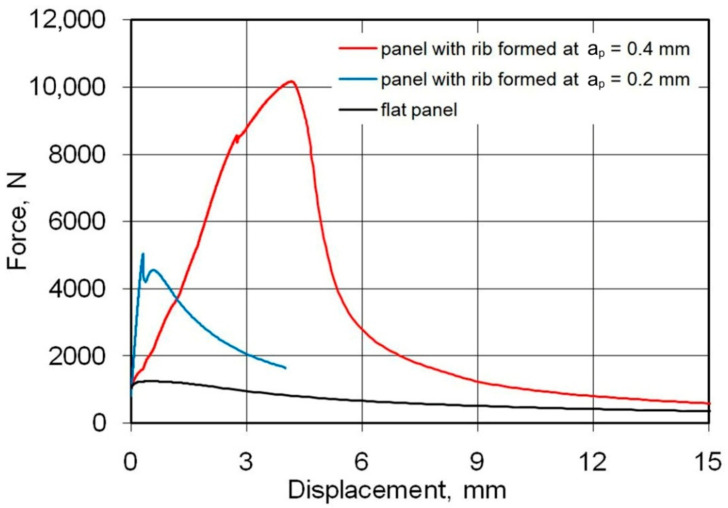
Load-displacement curves of the panels fabricated from 1-mm-thick EN AW-2024-T3 panels subjected to compression load.

**Figure 14:**
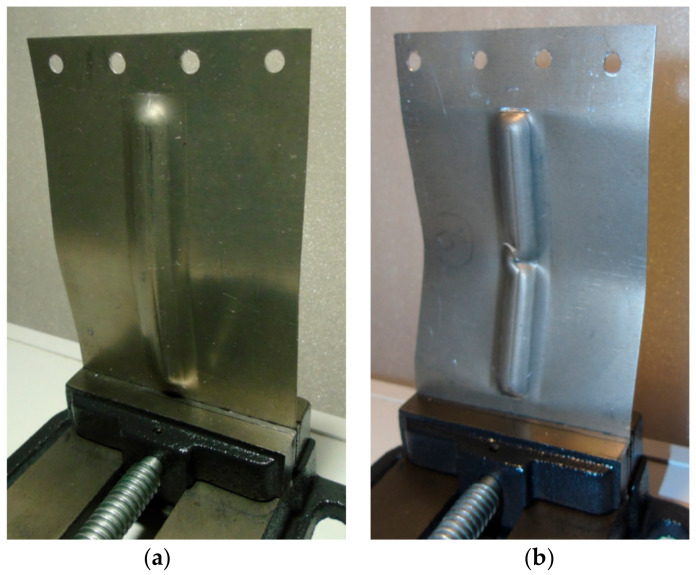
Buckling mode of 1-mm-thick EN AW-2024-T3 panel: (**a**) Mode A—the panels buckled halfway up the panel width towards the rib, without any appreciable loss of rib stability; (**b**) Mode B—the rib first lost stability at half height, which was associated with breakage of the rib, and then the panel was deflected in the opposite direction to the position of the rib.

Figure 13 shows the load-displacement curves of the panels fabricated from 1-mm-thick EN AW-2024-T3 sheets. The relationship of the buckling modes (Figure 14a,b) to the vertical pitch were the same as in the previously discussed damage modes of 0.4-mm-thick EN AW-2024-T3 Alclad and 0.8-mm-thick EN AW-7075-T6 Alclad sheets. Increasing the vertical pitch from 0.2 to 0.4 mm led to an increase in the lifting force from 5041 N to 10,068 N. A significant increase in the displacement of the grips was observed corresponding to the maximum force involved.

The occurrence of the two post-buckled shapes, denoted with A and B modes, is linked to different imperfections due to the fabrication process and with the asymmetry of the panel. What happens is common in similar structures and denotes imperfection sensitivity. This means that the structure can exhibit a different post-buckling behaviour dependent on small deviations of the data that are not necessarily due to the fabrication method, but can be related, for instance, to the loading (static, dynamic) or boundary conditions. One of many problems regarding the use of stringer-stiffened thin-walled load-carrying structures is the occurrence of various kinds of inaccuracies resulting from the method of applying the load (symmetrical, non-symmetrical) and, as mentioned above, the manufacturing process (geometric imperfections) [45,46].

### 3.2. Photogrammetric Analysis of Deformations

**Figure 15:**
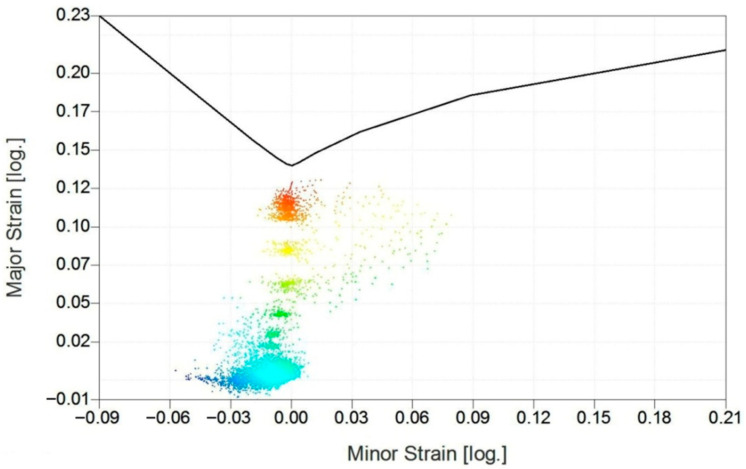
Forming limit diagram for a panel made of 1-mm-thick EN AW-2024-T3 sheet.

Information on the forming limit diagram for a specific grade of sheet material allows an assessment to be made of the risk of wrinkling failure and cracking [47,48]. Even if the crack is not visible on the rib surface, photogrammetric analysis of deformations permits non-destructive determination of the thinning of the sheet based on the surface deformation of the rib. Based on the results of photogrammetric measurements, the tool trajectory can be modified to ensure the desired uniform thinning of the sheet along the transverse profile of the ribs. An assessment of possible defects and failures is carried out on the basis of the location of the points corresponding to the actual material deformation in relation to the Forming Limit Curve (FLC) [49,50]. The FLC, as the key feature of the Forming Limit Diagram (FLD), records some pairs of in-plane limit strains (major and minor) and defines the boundary between the safe zone (no necking) and dangerous zone (necking and splitting), which is above the FLC. The forming limit curve shown in Figure 15 was determined using cupping tests on specimens of sheet metal by the Nakajima method. The FLC curve was measured and constructed by the Aramis system (GOM Gmbh, Braunschweig, Germany) according to ISO standard 12004-1:2020 [51]. Stochastic patterns were applied with a colour spray, and the shift of the patterns under load was evaluated. The final FLC was imported to the Argus system (GOM Gmbh, Braunschweig, Germany) and used for deformation analysis of the rib fabricated in 1-mm-thick EN AW-2024-T3 sheet metal.

In areas where the distribution of these deformations exceeds the forming limit curve, one can expect the occurrence of a specific defect in the drawpiece, i.e., a strong wrinkling trend, insufficient stretching, or cracks [52,53]. In order to maintain some safety margin, the deformation area boundary should not come too close to the forming limit curve. The forming limit diagram (Figure 15) plays an important role in the assessment of the suitability of the forming process [54,55,56]. All measurement points corresponding to deformation of the rib lie in the safe zone below the forming limit curve. However, some of the red points are above the value of 60% of major strain. One can speculate that increasing of the depth of the rib could damage the sheet metal.

**Figure 16:**
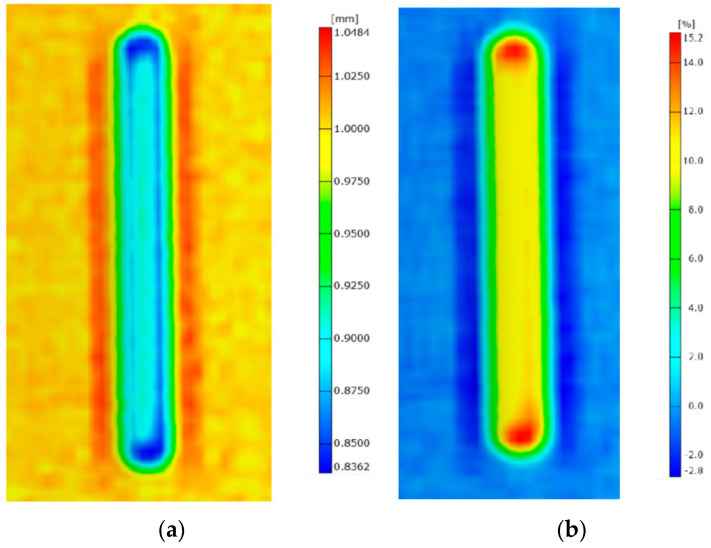
Distributions of (**a**) thickness and of (**b**) thinning of the rib fabricated in 1-mm-thick EN AW-2024-T3.

Measurements of the thickness distribution (Figure 16a) and thinning (Figure 16b) confirmed that the most stress-affected area in the panels is the corner of the rib, in which area the thinning exceeded 15.2%. Although the integrity of the material was not disturbed, this degree of thinning can be considered unsafe for the further forming of the rib. On the other hand, due to the very slender nature of the rib, the corner of the rib does not play an important role in the compression strength of the panels. The loss of stability was distributed to close to halfway up the height of the panels.

### 3.3. Fractographic Analysis of Fracture in the Panels

**Figure 17:**
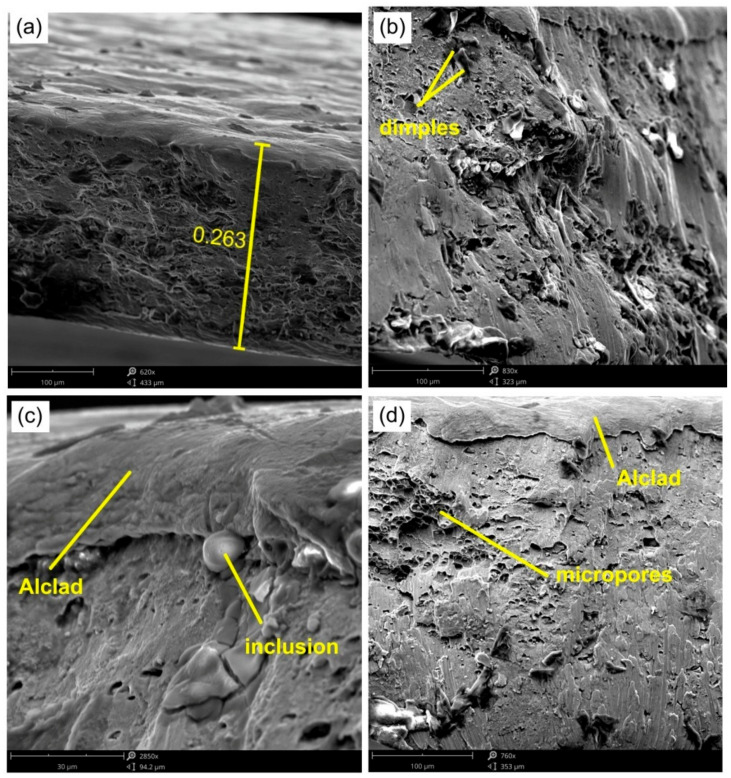
SEM micrographs of the fracture surface of a panel made of 0.5-mm-thick EN AW-2024-T3 Alclad sheet: Micrographs (**a**–**d**) show various fragments of the ductile fracture.

The fractographic analysis of the EN AW-2024-T3 samples showed the ductile nature of the destruction of the material as a result of the formation and joining of cracks preceded by plastic deformation. The crack in the sheet metal occurred in a plane perpendicular to its surface. Figure 17 shows SEM micrographs of the fracture surface of the sample shown in Figure 10b.

The formation of ductile fracture along the slip planes during the development of a ductile fracture is preceded by large deformations of the material in front of the crack. Under these conditions, the thickness of the sheet metal at the crack formation site was 0.263 mm (Figure 17a), so the failure of the base material (EN AW-2024-T3) was preceded by a percentage relative deformation of the sheet through a thickness of approximately 30%.

The thickness of Alclad applied to the surface of aluminium alloys intended for aviation applications is approximately 5% for sheets with a thickness below 1.57 mm and 2.5% for sheets with a thickness above 1.57 mm. The Alclad material consists of a very large amount of pure aluminium which is very plastic. The fracture surface of the Alclad zone is much smoother than the surface of the base material (Figure 17c,d). Ductile cracking occurs by the nucleation and expansion of voids, and is usually initiated around inhomogeneities or from particles of a different phase. The microstructure of the EN AW-2024-T3 alloy contains not only the α and Θ phases, but also the Θ’ precipitates, as shown by Huda et al. [56]. The α phase corresponds to the dissolution of copper and other alloying elements in the aluminium crystal lattice [57]. The Θ phase corresponds to the intermetallic compound CuAl2 [58,59]. The Θ phase appears as a colony of precipitates in the microstructure, while the Θ’precipitates are evenly distributed throughout the microstructure [56]. The presence of these components in the EN AW-2024-T3 alloy results from its production process, i.e., heat treatment in a bath, cold plastic working, and natural aging [60].

**Figure 18:**
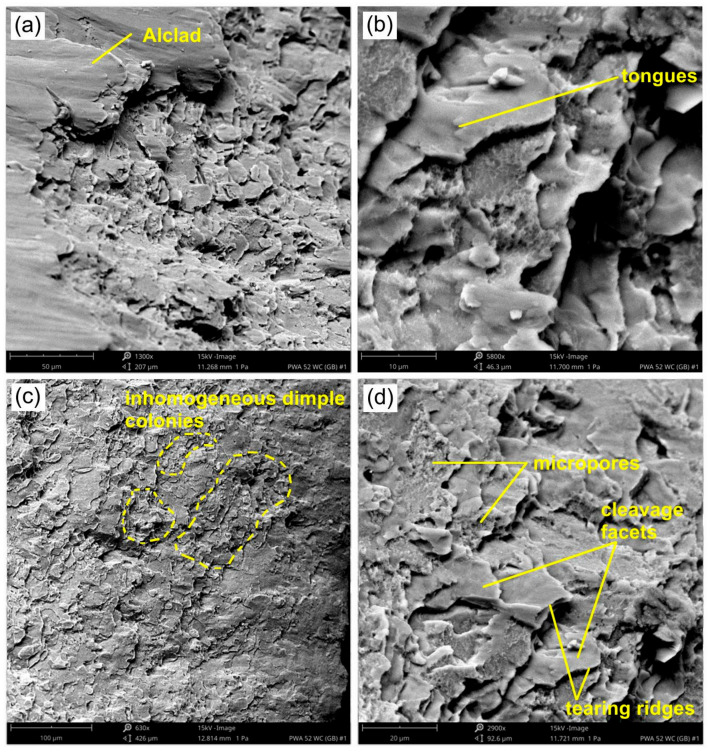
SEM micrographs of the fracture surface of a panel made of 0.8-mm-thick EN AW-7075-T6 Alclad sheet: Micrographs (**a**–**d**) show various fragments of the ductile fracture.

The mechanism of fracture of the 0.8-mm-thick EN AW-7075-T6 Alclad is similar. The sheet thickness at the area of the crack initiation was 0.82 mm. The fracture first takes place in the inner part of the fracture front and then expands towards the outer side of the rib. At the edge of the sheet, the crack propagates along the shear planes, thus creating shear lips [61,62]. In turn, in the central part of the material the fracture process is dominated by the nucleation and development of voids. The different value of deformations between the matrix and hard particles distributed in the matrix causes the generation of dislocations in the matrix and intensification of the work hardening phenomenon. On the other hand, if brittle particles are arranged in the matrix, they are not able to accommodate large plastic deformations, in contrast to the matrix. Under such conditions, decohesion may occur at interfacial surfaces [63]. The generation of micro-voids on inclusions leads to the local formation of necks between the micro-voids, the breaking of which leads to the joining of voids formed in the microparticles. The micro-voids on the fracture surface were irregular and of various sizes (Figure 18b,c). In the internal side of the rib, the pores are more developed, contrary to the outer layer. There is therefore a strong relationship between local strain hardening and the action of the rounded tip of the tool on the inner surface of the rib.

## 4. Conclusions

The following conclusions are drawn from the experimental research on (i) panel fabrication and (ii) axial compression tests:

- Vertical pitch influences the value of transverse and longitudinal deflection as a result of the springback phenomenon.
- Higher deflection in both longitudinal and transverse directions results in the destruction of the panel dependent on the buckling mode A.
- Lower both longitudinal and transverse deflection results in the destruction of the panel according to buckling mode B.
- The load-capacity of the panels buckled in mode B was higher at 52%, 123%, and 99% in the case of 0.4-mm-thick EN AW-2024-T3 Alclad sheet, 0.8-mm-thick EN AW-7075-T6 Alclad sheet, and 1-mm-thick EN AW-2024-T3 sheet, respectively, compared to the load-capacity of the panels buckled in mode A;
- Examination of the morphologies of the fracture surfaces showed the ductile nature of material failure as a result of the formation and joining of cracks preceded by plastic deformation.

## Figures and Tables

**Table 1:** Chemical composition of test sheets in wt.% [40].

Alloy	Si	Fe	Cu	Mn	Mg	Cr	Zn	Ti	Other Elements		Al
									Each	Total	
2024-T3	0.50	0.50	3.8–4.9	0.3–0.9	1.2–1.8	0.10	0.25	0.15	0.05	0.15	remainder
7075-T6	0.40	0.50	1.2–2.0	0.30	2.1–2.9	0.18–0.28	5.1–6.1	0.20	0.05	0.15	remainder

**Table 2:** Basic mechanical properties of test sheets [40].

Material	Temper	Specified Thickness, mm		Tensile Strength R_m_, MPa		Yield Stress R_p0.2_, MPa		Elongation A_50_ min., %
		over	through	min.	max.	min.	max.	
EN AW-2024	T3	0.50	3.20	435		290		15
EN AW-2024 Alclad	T3	0.25	0.50	405		270		12
EN AW-7075 Alclad	T6	0.32	1.00	490		420		8

## Data Availability

The data presented in this study are available on request from the corresponding author.
